# Fear of drowning (thalassophobia) and its coping strategies in nurses working in public hospitals in Eastern Guilan 

**Published:** 2022-01

**Authors:** Shiva Mahdavi Fashtami, Azar Darvishpour

**Affiliations:** ^*a*^ Master of Internal-Surgical Nursing, Pirouz Hospital, Guilan University of Medical Sciences, Rasht, Iran.; ^*b*^ Assistant Professor of Nursing, Zeynab (P.B.U.H) School of Nursing and Midwifery, Guilan University of Medical Sciences, Rasht, Iran.; ^*c*^ Social Determinants of Health (SDH) Research Center, Guilan University of Medical Sciences, Rasht, Iran.

## Abstract

**Background:**

Thalassophobia is a special type of fear that is a constant and intense fear of deep water such as the ocean or sea. The aim of this study was to identify the fear of drowning (thalassophobia) and its coping strategies in nurses working in public hospitals in the east of Guilan province in 2021.

**Methods:**

This is a cross-sectional descriptive study in which 156 nurses working in public hospitals in East of Guilan province participated by convenience sampling. The research tool was the thalassophobia Questionnaire and Coping Strategies which were designed online and made available to participants through virtual networks. Descriptive statistics were used to analyze the data using online questionnaire system.

**Results:**

The majority of the samples (37%) were in the age group of 50-41. In terms of gender, 99% of all participants were female, married (78.5%) and had a bachelor' degree (85%). Most of them (31%) had 11-15 years of work experience and the majority (61.3%) were officially employed in terms of employment status. Regarding the items, majority of the participants stated that they were afraid of deep water (83.8%), when they go to deep places, they have shortness of breath (83.8%), they cannot swim alone (42.5%), they are afraid of drowning in the ocean more than anything there (61.3%). Despite these results, which are in favor of diagnosing thalassophobia, the majority answered that they were willing to travel by ship (61.3%) or if traveling by ship was the only option, they could board it (43.8%). Concerning the coping strategies, participants used firstly emotion-focused strategies (52.6%) and secondly, avoidance-based strategies (43.8%).

**Conclusions:**

Although dealing with thalassophobia is challenging, there are ways to cope and reduce fear, and relaxation strategies can be used to calm the mind and body.

**Keywords:**

Drowning, Fear, Thalassophobia, Coping Strategies

